# Recent Advances in Zinc Hydroxystannate-Based Flame Retardant Polymer Blends

**DOI:** 10.3390/polym14112175

**Published:** 2022-05-27

**Authors:** Wei-Hao Pan, Wen-Jie Yang, Chun-Xiang Wei, Ling-Yun Hao, Hong-Dian Lu, Wei Yang

**Affiliations:** 1 School of Energy, Materials and Chemical Engineering, Hefei University, Hefei 230601, China; pwh971007@163.com (W.-H.P.); weicx@hfuu.edu.cn (C.-X.W.); luhdo@hfuu.deu.cn (H.-D.L.); 2 Department of Architecture and Civil Engineering, City University of Hong Kong, 88 Tat Chee Avenue, Kowloon, Hong Kong, China; wenjiyang8-c@my.cityu.edu.hk; 3 School of Materials Engineering, Jinling Institute of Technology, Nanjing 211169, China; hly@jit.edu.cn

**Keywords:** zinc hydroxystannate, polymer composites, flame retardant, smoke suppression

## Abstract

During the combustion of polymeric materials, plenty of heat, smoke, and toxic gases are produced that may cause serious harm to human health. Although the flame retardants such as halogen- and phosphorus-containing compounds can inhibit combustion, they cannot effectively reduce the release of toxic fumes. Zinc hydroxystannate (ZHS, ZnSn(OH)_6_) is an environmentally friendly flame retardant that has attracted extensive interest because of its high efficiency, safety, and smoke suppression properties. However, using ZHS itself may not contribute to the optimal flame retardant effect, which is commonly combined with other flame retardants to achieve more significant efficiency. Few articles systematically review the recent development of ZHS in the fire safety field. This review aims to deliver an insight towards further direction and advancement of ZHS in flame retardant and smoke suppression for multiple polymer blends. In addition, the fire retarded and smoke suppression mechanism of ZHS will be demonstrated and discussed in depth.

## 1. Introduction

Currently, polymers, including plastics, rubbers, and fibers, are manufactured in large quantities and are widely used in industry, civil, aerospace, and other fields [1,2,3]. However, most polymeric materials are flammable. When ignited, lots of heat, soot, and toxic gases are released, which are extremely harmful to human beings and cause enormous environmental pollution [4,5]. To address these issues, varied flame retardants have been developed, such as phosphorus- [1,6,7], and halogen-based compounds [6,7] and nanomaterials, (e.g., carbon nanotubes [8,9], graphene [10,11], MXene [12]), etc. These fillers can efficiently improve the flame retardant properties of polymers and reduce the heat release during combustion. Nevertheless, one issue that attracts enormous attention is the inability to suppress the emission of smoke generated from the burning of polymers. The smoke is the primary risk factor for people’s lives in fires, which seriously delays the opportunity to escape and save property. Especially for some polymers, including polyvinyl chloride (PVC), acrylonitrile-butadiene-styrene co-polymer (ABS) and epoxy resins (EP), smoke suppression is more critical than flame retardancy. Therefore, smoke suppression agents have received extensive attention from scientific and industrial researchers.

The commonly used smoke suppressants are molybdenum oxides [13,14], iron-, [15,16], boron- [17,18] and tin-based compounds [19]. Among them, tin-based compounds are a new kind of environmentally friendly smoke suppressant (Table 1). Zinc hydroxystannate (ZHS, ZnSn(OH)_6_), a typical perovskite-shaped hydroxy compound, is a novel type of high-efficiency, environmentally friendly flame-retardant and smoke suppressant [20,21,22]. It was utilized for enhancing the flame retardancy and smoke suppression of polymer by Cusack and Hornsby thirty years ago [23,24,25]. In addition, it was demonstrated that ZHS can be used as a substitute for Sb_2_O_3_ as a synergistic flame retardant with halogenated flame retardants, contributing to environmental protection as a non-toxic flame retardant [26,27]. In the future, the trend of product functionalization, miniaturization, replacing steel with plastic, and people’s increased recognition of flame retardant concepts and the pursuit of green environmental protection, will enable tin-based flame retardants to be used in a wider range of applications.

To date, several review articles corresponding to fire-retarded polymer composites have been published [28,29,30]. However, few papers focusing on the utilization of ZHS in flame retardant polymer blends have been reported. This article summarized the preparation and modification approaches of ZHS. The flame-retardant and smoke suppression properties of ZHS in various polymers were introduced, and the mechanisms were discussed in depth. Moreover, we presented the prospect and pointed out the future development direction of ZHS.

## 2. Preparation of ZHS-Based Flame Retardant

### 2.1. Hydrothermal Method

The hydrothermal synthesis method refers to utilizing the high temperature and high-pressure conditions to promote the chemical synthesis reaction of the raw materials in the aqueous solution [31]. Sun et al. [32] employed zinc acetate dihydrate (Zn(CH_3_COO)_2_ 2H_2_O) and tin chloride pentahydate (SnCl_4_ 5H_2_O) as raw materials to prepare ZHS via hydrothermal method. After centrifuging with distilled water and ethanol, ZHS with a diameter of 420 nm was obtained. Zhan et al. [33] prepared single-crystalline ZHS microcubes on indium tin oxide (ITO) glass substrates by using a hydrothermal growth method. They coated ITO substrate with zinc acetate dihydrate (Zn(CH_3_COO)_2_ 2H_2_O) solution and annealed at 300 °C for 10 min to obtain ZnO coated ITO substrate. After treatment via a hydrothermal process with SnCl_4_ 4H_2_O solution, the microtube of ZHS was obtained with an edge length of 5–8 mm. Qin et al. [34] used α-{Cu, Sn} copper foils to synthesize a single crystalline via a low-temperature hydrothermal method. The obtained ZHS nanoparticles showed cubic perovskite crystalline structure and uniform arrangement on the substrate. Jose et al. [35] prepared novel skeleton-like and rod-shaped ZHS nanoparticles by means of a simple hydrothermal approach. f-Zn(NO_3_)_2_-6H_2_O and SnCl_4_-5H_2_O were used as raw materials. The nanomaterials were prepared with diameters ranging from 12–18 nm. Fan et al. [36] synthesized novel hollow core–shell ZHS microspheres at room temperature by using NH_3_ bubble templating technique via a hydrothermal recrystallizing process. During the formation of ZHS, NH_4_F that was added into the solution reacted with NaOH to form NH_3_, which immediately reacted with Zn^2+^ to generate Zn(NH_3_)_4_^2+^ complexes. During the reaction, NH_3_ gas bubbles were produced to attach to the surface of ZnSn(OH)_6_ nanoparticles which aggregated to minimize interfacial energy. Subsequently, driven by the depreciation of interfacial energy, ZnSn(OH)_6_ nanoparticles migrated along the surface of NH_3_. The bubbles continued to grow and produce a hollow core–shell ZnSn(OH)_6_ microsphere.

### 2.2. Precipitation Method

The preparation of ZHS via precipitation primarily includes two strategies: co-precipitation and homogeneous precipitation. Co-precipitation is one of the most commonly used methods for fabricating ZHS at nanoscale. It is prepared by dissolving the electrolyte of each component and adding a suitable precipitating agent to form a precipitate with drying and grinding. The process conditions of this method are easy to control with short production cycle and low cost, which is suitable for industrial batch production [37]. Yang et al. [38] mixed SnCl_4_ 5H_2_O and ZnCl_2_ in a certain proportion to form a solution by using an alkaline solution to adjust the pH of the solution to more than 9. The precipitate was produced in the solution, and ZHS was gained after washing and drying. Kramer et al. [39] introduced microwave into the chemical co-precipitation by adding a certain ratio of zinc chloride and sodium stannate configuration into a mixed solution. A quartz crucible containing the precipitate was placed in the microwave oven reaction for 10 min; the precipitate was collected and dried to obtain ZHS powder. Another method is the homogeneous precipitation route. Homogeneous precipitation refers to the planned and controlled generation of ZHS nanoparticles in chemical solutions. For example, Chen et al. [40] used ZnCl_2_ and SnCl_4_ 5H_2_O as raw materials and prepared a solution by mixing NaOH with ZnCl_2_. The precipitation was achieved via strong stirring. In contrast to ordinary precipitation, homogeneous precipitation allows the large-scale preparation with well-structured ZHS particles by using suitable precipitants such as urea. 

### 2.3. Others

In addition to the common methods, other approaches have been employed to prepare ZHS particles, including cationic membrane electrolysis [41], mechanochemical synthesis [42], chemical conversion [43], and self-templated synthesis [44]. Onwudiwe et al. [45] used Zn(ac)_2_ 2H_2_O and SnCl_2_ 2H_2_O as raw materials to synthesize ZHS with the assistance of microwave. ZHS was obtained after purification and drying of the product prepared by repeated heating in a microwave oven by employing ethylene glycol as the solvent. Xu et al. [46] adopted zinc acetate (Zn(CH_3_COO)_2_ 2H_2_O), tin tetrachloride (SnCl_4_ 5H_2_O), and monoethanolamine to prepare precursor solution. After aged for 24 h, the upper layer was collected for the sol solution. The deposition process was carried out on the S substrate rotated at 1000 rpm rotation and 2500 rpm rotation for 10 s and 20 s, respectively, and followed by a heating process. The process repeated by 8 times to obtain the entire ZHS film. Xu et al. [47] utilized the liquid laser ablation method to fabricate ZHS nanoparticles. They used the first harmonic of an Nd:YAG (yttrium aluminum garnet) laser to irradiate the Zn plate which was fixed on the bracket in water. After irradiation for 20 min, the ZnO colloidal solution reacted with Na_2_SnO_3_, which resulted in the production of ZHS. Nikolic et al. [48] prepared ZHS nanoparticles via mechanical activation. They mixed ZnO and SnO_2_ powders with grinding in a vibro-mill to produce ZHS. 

In summary, hydrothermal, precipitation, and other methods present their advantages. Compared with the high-temperature reaction (hydrothermal method), the precipitation shows the benefits of good dispersibility, easy control of particle size, and high purity. However, due to the limitation of the preparation strategy, the hydrothermal and precipitation methods are still the primary methods for producing ZHS in the industry at present. Alternatively, the obtained products have disadvantages such as single morphology and severe agglomeration. Therefore, how to realize the controllable preparation of ZHS is a problem that needs to be solved in the future.

## 3. The Application of ZHS-Based Flame Retardant

ZHS is a highly effective and environmentally friendly flame retardant and smoke suppression additive in various polymers. However, pure ZHS shows a limited flame retardant effect and often needs to be combined with other flame retardants to achieve the optimal fire retarded and smoke suppression efficiency. ZHS-based flame retardants are primarily utilized in PVC, EP, and other polymers for improving fire safety properties.

### 3.1. In PVC

PVC is commonly fabricated by polymerizing vinyl chloride monomer. It is widely used in construction, industrial products, sealing materials, and fibers. PVC has excellent flame retardant properties. However, PVC contains a large number of chlorine atoms, and lots of smoke, toxicity, and corrosive gases are produced when burning, which are extremely harmful to human beings. Therefore, suppressing smoke and toxicity is a big challenge.

Xu et al. [49] compared the flame retardancy and smoke suppression properties of inorganic tin compounds such as ZHS and zinc stannate (ZS) with alumina trihydrate, magnesium hydroxide, and Sb_2_O_3_. The results showed that ZHS and ZS can be used as highly efficient flame retardants for flexible PVC, which was mainly in the form of Lewis acid for flame retardant action. The presence of ZS and ZHS can catalyze the early cross-linking of PVC, thus enhancing the LOI and char yield of the samples. Sn primarily presented in the residual char of the PVC samples treated with tin oxide in the form of stannous oxide and tin chloride, which also demonstrated that tin oxide acted in the condensed phase.

Other flame retardants are often required to contribute to the synergistic effect to achieve optimal flame retardant efficiency. For example, Qu et al. [50] prepared melamine hydroxystannate (MASN) and a composite of MASN and ZHS (MAZSN) to improve the flame retardant properties of PVC. Among them, PVC was used as carbonizing agent, and Sn(OH)_2_ and Zn^2+^ were used as acid sources in flame retardants that can catalyze the early crosslinking of PVC compounds. In addition, hydrogen chloride and melamine can promote the formation of carbon residue. On the other hand, the released hydrogen chloride can act as an effective gas-phase flame retardant, and metal ions can promote the stability of carbon residues.

**Figure 1:**
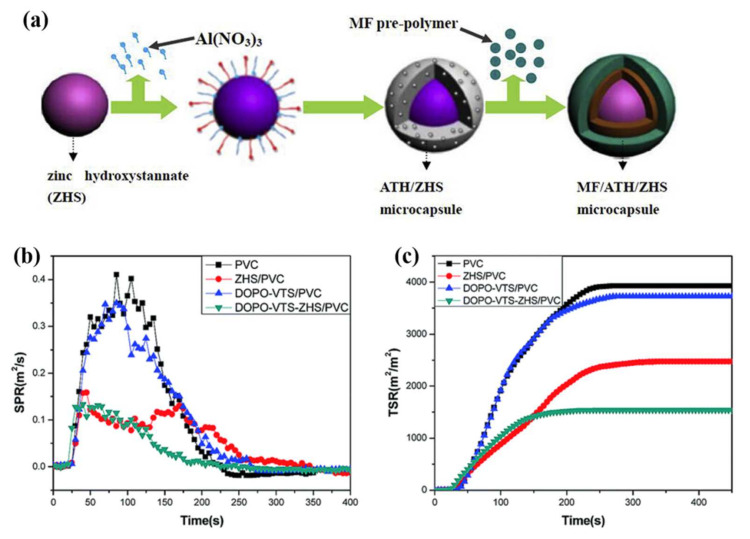
(**a**) The synthesis process of MF/ATH/ZHS, reproduced with permission from reference [54] copyright (2017) Elsevier. HRR (**b**) and THR (**c**) versus time curves of PVC nanocomposites during combustion, reproduced with permission from reference [56] copyright (2015) The Royal Society of Chemistry.

Metal oxides and hydroxides that show good flame retardant performance, are also utilized with ZHS to contribute synergistic flame retardant effect in PVC [51,52]. Yang et al. [38] synthesized ZHS precipitates on the surface of magnesium hydroxide and calcium carbonate. The addition of the inorganic fillers into PVC significantly reduced the peak heat release rate (PHRR) with a 34% reduction. Qu et al. [53] prepared ZHS-modified alumina trihydrate (ATH) or magnesium hydroxide (MDH) to improve the LOI value and char residues of PVC. During combustion, the elemental zinc and SnO_3_^2−^ in the two composite flame retardant materials reacted with the HCl produced by the decomposition of PVC to release ZnCl_2_, SnCl_2,_ and H_2_O, which were effective catalysts for the cross-linking of PVC into char as Lewis acids. The resulting char layer showed a denser structure which effectively inhibited combustible gases and transfer of heat to the PVC. Zhang et al. [54] prepared aluminum hydroxide (ATH)/zinc hydroxystannate (ZHS) microcapsule using the sol–gel method, and prepared dimeric formaldehyde resin (MF)/ATH/ZHS microcapsules for enhancing the flame retardant and smoke suppression of PVC (Figure 1a). MF/ATH/ZHS microcapsule presented uniform dispersion in the PVC matrix, leading to the improvement of tensile strength, elongation at break, flame retardancy, and reduction in smoke density compared to PVC/ATH/ZHS microcapsule [55]. After adding the microcapsules of 10 g 2/8ATH/ZHS, 10 g 5/5ATH/ZHS, 10 g 8/2ATH/ZHS, 10 g 2.5/2/8MF/MF/ATH/ZHS, 10 g 10/2/8/MF/ATH/ZHS, 10 g 40/2/8MF/ATH/ZHS to PVC, the LOI values of the PVC composites increased to 29.3%, 29.1%, 28.1%, 31.2%, 29.4%, and 29.3%, respectively. The smoke density values of the PVC composites decreased to 45%, 43%, 68%, 32%, 33% and 35%, respectively. The ZHS showed a significant increase in the fire resistance of PVC, as these capsules effectively prevented the heat and oxygen entering PVC. In addition, during combustion, PVC composites with ATH/ZHS and MF/ATH/ZHS microcapsules underwent pyrolysis, resulting in the release of water vapor and NH_3_ from MF to reduce the air concentration, causing the PVC material to expand into char. What is more, the presence of Al-Sn-Zn and N-Al-Sn-Zn functional groups on the microcapsules endowed them with specific surface properties that contributed to the flame and smoke retardation.

In addition, other inorganic materials have been developed to enhance the flame retardancy of PVC by combining with ZHS. For example, ZHS, MgSn(OH)_6_, SrSn(OH)_6_ were used to modify CaCO_3_ ((ZHS-MgSn(OH)_6_-coated CaCO_3_, ZHS-SrSn(OH)_6_-coated CaCO_3_)) to enhance the flame retardancy of PVC [57]. The hydroxystannate coating of CaCO_3_ promoted its dispersion, which resulted in a more significant flame retardant effect with improved LOI values and char yields in PVC. Jiao et al. [58] employed cetyltrimethylammonium bromide (CTAB)/aqueous glycerol solution as a mild reaction environment to control the crystallization of CaCO_3_ and ZHS to obtain ZHS-coated dendritic fibrous CaCO_3_. These composites exhibited higher thermal stability and flame retardant properties than ZHS-coating cubic CaCO_3_. The mechanical properties in terms of tensile strength, elongation at break, and impact strength were increased by 0.4–2.8 MPa, 7–25%, and 11–20%, respectively. Liu et al. [59] used a simple ultrasound-assisted method to gain ZHS-coated dendritic fibrous barium carbonate (ZHS/BaCO_3_-F). It was found that the optimal overall performance of the PVC was achieved at 5 wt% addition, with the LOI value increasing from 27.0% to 30.2% and the remained tensile strength.

Gao et al. [60] prepared ZHS-modified graphene oxide (GO) which was added to flexible PVC. The PHRR and total heat release (THR) values of ZHS/GO/PVC were significantly reduced by 50% and 59.7%, respectively, compared to pure PVC due to the synergistic effect of ZHS and GO. The amount of organic volatiles released during the combustion of PVC was remarkably reduced by adding ZHS/GO. Huo et al. [61] synthesized a chitosan-modified zinc hydroxystannate (ZHS-CS) by using a cationic and anionic substitution strategy. They mixed ZHS-CS with reduced graphene oxide (rGO) to synthesize a novel flame retardant (ZHSCS/rGO) for PVC. By replacing one-fifth of the zinc ions in ZHS with chitosan cations, Sn-4Zn-1CS/rGO was obtained. It was found that ZHSCS/rGO could improve the performance of PVC composites, and the total heat and smoke emissions of PVC/Sn-4Zn-1CS/rGO were reduced by 24.2 and 40.0%, respectively, compared with pure PVC. Gao et al. [56] used ZHS to coat an organophosphorus flame retardant to form a composite material with a synergistic organic/inorganic flame retardant. When 5 wt% DOPO-VTS-ZHS was added into PVC, the PHRR and THR were significantly reduced by 39% and 50%, and the LOI value increased from 25.8% to 30.2%. Additionally, the smoke production rate (SPR) and total smoke release (TSR) of DOPO-VTS-ZHS/PVC also showed a great reduction compared with pure PVC, which indicated the great smoke-suppressing property of DOPO-VTS-ZHS (Figure 1b,c). Sang et al. [62] prepared zinc hydroxytitanate nanotubular (ZHS-TNT) flame retardants by using a facile in situ solution method to inhibit the smoke emission during the combustion of PVC. When ZHS-TNT was added at 2.5 wt%, the LOI value increased from 25.8% to 29.6%, and the total smoke emission and average specific extinction area were reduced by 40% and 34%, respectively.

### 3.2. In EP

EP is a versatile thermosetting material with excellent properties such as excellent adhesion, mechanical properties, and chemical resistance which is used in many applications including optical machinery, railway vehicles, aerospace industry, and solar cells. However, EP is highly flammable, posing a high fire risk to people’s lives and properties. Thus, it is required to improve the fire safety performance of EP. Similar to PVC, ZHS is commonly used as a smoke suppressing compound that combines with various flame retardants to enhance the fire safety properties of EP.

Nanoparticles can effectively prevent the migration of heat and oxygen during combustion due to their nano-effect. Accordingly, studies on the synergistic flame retardant effect between nanoparticles with ZHS have been reported. Zhang et al. [63] synthesized layered bimetallic (Ni-Co) hydroxides (NCH) and encapsulated the ZHS nanotubes in this 3D nanocage (ZHS@NCH). The addition of 6 wt% ZHS@NCH increased the LOI value of the nanocomposites to 27.2% with a UL-94 V-0 rating. At the same time, the PHRR and total smoke production (TSP) were reduced by 69.1% and 36.1%, respectively, compared to pure EP. The catalytic effect of zinc and tin influenced the decomposition path of the epoxy, promoting early cross-linking and char formation. The decomposed metal elements migrated to the polymer surface during combustion and catalyzed the formation of robust chars. Furthermore, the transition metal elements Ni and Co in layered bimetallic Ni–Co hydroxides (NCH) showed a positive effect on the enhancement of char formation than Zn and Sn. The homogeneous distribution of ZHS in the hollow NCH nanocages allowed the hybrids to interact with the polymer matrix in the interface and catalyze the char formation in the neighboring polyhedral. In addition, the decomposition of hydroxyl groups in the ZHS and NCH, as well as the release of water and anions from the NCH interlayer, diluted the flammable volatiles in the flame zone and also acted as a flame retardant in the gas phase. To enhance the flame retardant and smoke suppressive properties of EP, ZHS@Mg-Al-LDH and ZHS@α-ZrP were prepared by co-precipitation method through electrostatic interaction, which was added into EP [64]. The LOI of ZHS@Mg-Al-LDH/EP and ZHS@α-ZrP/EP presented an enhancement of 25.7% and 26.0% compared with neat EP. The PHRR was significantly reduced by 48.2% and 47.7% as well as the SPR by 21.6% and 27.1%, respectively, compared to pure EP. The barrier effect of Mg-Al-LDH and α-ZrP inhibited the heat emission due to its special structure. Additionally, the catalytic effect of ZHS contributed to the formation of a char layer, where various metal oxides formed during combustion were deposited on the surface of the char layer. It effectively improved the thermal stability of the residual char and inhibited the release of smoke during combustion. Wang et al. [65] prepared ZHS doped titanium carbide (Ti_3_C_2_T_x_) MXene nanosheets (ZHS@M) for improving the flame retardancy of EP. Due to the 0D-2D hierarchical structure of ZHS@M, the PHRR and THR of EP decreased by 54.91% and 58.74%, respectively. Moreover, toxic gases (CO and CO_2_) were decreased by 44.44% and 39.46% with the addition of 2 wt% ZHS@M. ZHS@M also improved the mechanical property of EP (tensile robustness 64.71 MPa and modulus 2.39 GPa).

**Figure 2:**
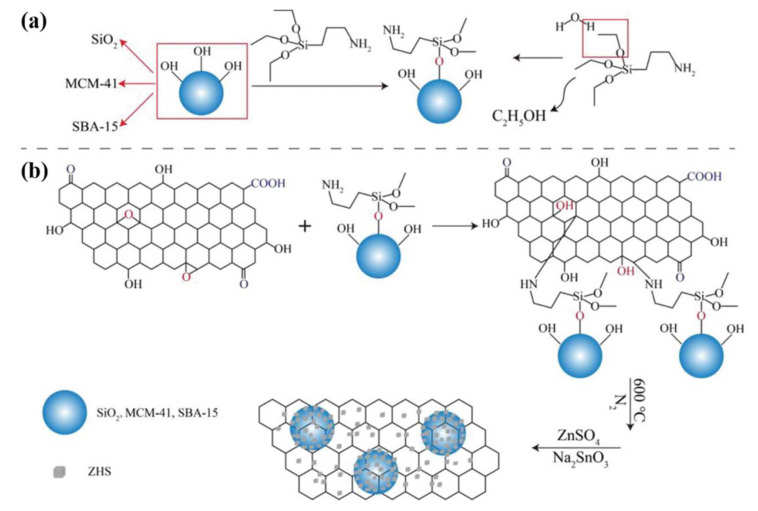
(**a**) The modification of SiO_2_, MCM-41 and SBA-15; (**b**) The preparation of SiO_2_-RGO-ZHS, MCM-41-RGO-ZHS, and SBA-15-RGO-ZHS, obtained from permission from reference [66] copyright (2020) John Wiley and Sons.

**Figure 3:**
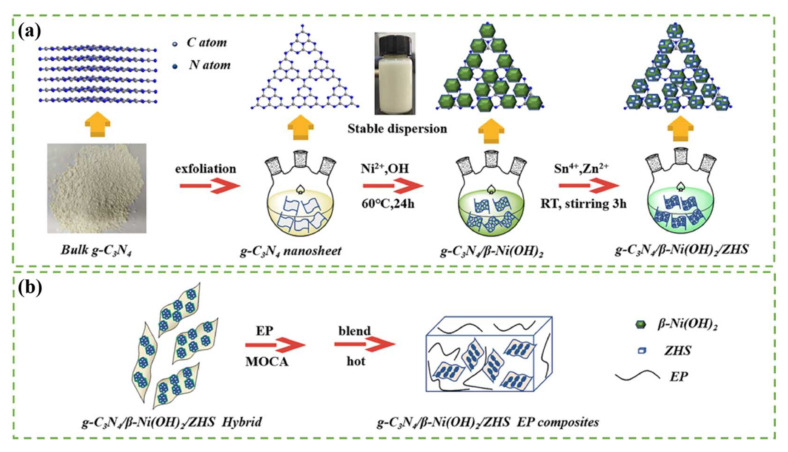
The preparation process of (**a**) g-C_3_N_4_/β-Ni(OH)_2_/ZHS and (**b**) EP composite, obtained from permission from reference [68] copyright (2020) Elsevier.

Wang et al. [66] successfully prepared zinc hydroxystannate (ZHS)-mesoporous silica (SBA-15 and MCM-41) modified RGO, which was used to improve the fire safety of EP (Figure 2). SBA-15-RGO-ZHS/EP showed the lowest TSP (22.8 m^2^) and PHRR (416 kW m^−2^) with the highest LOI value (29.4%) compared to pure EP. Li et al. [67] covalently grafted polyetheramine (M2070) onto the surface of graphene nanosheets with the subsequent modification of ZHS to obtain a fluidic type of organic hybrid material (GNS-ZHS-M2070). The unique fluidity of the organic hybrid material and the soft organic shell provided excellent processing properties as well as good compatibility in EP. When GNS-ZHS-M2070 was added at 2%, the PHRR value decreased to 561.2 kW m^−2^ with a reduction of 21.8% compared to pure EP (718 kW m^−2^). When the loading increased to 12%, the PHRR value decreased to 470.6 kW m^−2^ with a reduction of 34.5%. Xu et al. [68] prepared nickel hydroxide and ZHS double-modified graphitic carbon nitride (g-C_3_N_4_/β-Ni(OH)_2_/ZHS) (Figure 3). When it was added at 3 wt%, the peak exothermic rate, total exothermic rate, SPR, and TSP of the composite decreased by 39.2%, 15.5%, 39.2%, and 14.2%, respectively, compared to EP. During combustion, metal oxides containing Ni, Zn and ZnSnO_3_ were produced from the decomposition of g-C_3_N_4_/β-Ni(OH)_2_/ZHS, which promoted the formation of char layers. In addition, the barrier effect of the g-C_3_N_4_ nanosheets effectively prevented the pyrolysis products from entering the gas phase, further increasing the amount of char formation, indicating a reduction in volatile combustible gases. Liu et al. [69] synthesized ZHS/RGO hybrids via a hydrothermal method to reduce the fire hazards of EP. The cone calorimetry results showed that the incorporation of 3.0% ZHS/RGO led to significant reductions in PHRR and THR by 50% and 39%, respectively.

Wang et al. [70] prepared ZHS on the surface of amorphous hydrous TiO_2_ solid spheres (AHTSS) by a layer-by-layer self-assembly method. The TGA results of the EP composites showed that the addition of AHTSS@PEI@ZHS led to a higher yield of char residues than that of AHTSS and ZHS alone. The introduction of AHTSS@PEI@ZHS to EP exhibited good flame retardancy and smoke suppression properties. Liu et al. [71] fabricated MnO_2_@ZHS hybrid materials by using a simple electrostatic adsorption method. After adjusting the pH of the ZHS suspension to 5 with 0.1 M hydrochloric acid, the suspension was slowly dropped into the MnO_2_ solution and stirred thoroughly to obtain the desired MnO_2_@ZHS binary hybrid. It acted with a strong interfacial interaction with the EP, which was thus well dispersed in EP, resulting in improved flame retardancy. The HRR value gradually decreased with the increase in MnO_2_@ZHS addition, and the THR value decreased by nearly 40% compared with pure EP. Due to the catalytic effect of manganese dioxide, the pre-decomposition of EP and the degradation of metal oxides were promoted, and the MnO_2_@ZHS binary hybridization can promote the density and graphitization degree of carbon residues during combustion, thereby delaying the penetration of oxygen and combustible gases. Gao et al. [72] used 2-carboxyethylphenylphosphoric acid (CEPPA) to modify ZHS and successfully synthesized the CEPPA–ZHS hybrid via a solution approach. After adding 10 wt% of CEPPA-ZHS into EP, the char residues and LOI values of CEPPA-ZHS/EP were significantly increased. Moreover, the cone calorimeter data showed that the PHRR and THR were decreased by ca. 45% and 20.4%, respectively, due to the synergistic effect of CEPPA and ZHS which can promote the formation of chars on the surface of EP to reduce the flammability. Accordingly, the heat and mass transfer between the gas phase and condensed phase was retarded. The decrease in smoke emission was attributed to the barrier effect of ZHS that was enhanced by CEPPA. 

### 3.3. In Other Polymers

Except for PVC and EP, ZHS-based flame retardant have also been employed in the other polymers such as ABS [73], poly(acrylonitrile-co-vinylidene chloride) (PANVDC) [74], ethylene-vinyl acetate copolymer (EVA) [75], polypropylene/ethylene propylene diene [76], polyethylene terephthalate (PET) [77], etc.

Cusack et al. [26] investigated the smoke suppression property of ZHS and antimony trioxide (Sb_2_O_3_) in halogenated polyester resin. The results showed that ZHS was more efficient than Sb_2_O_3_ in reducing PHRR in chlorinated and brominated resin. The PHRR of the chlorinated and brominated resin was reduced by 38% and 39%, respectively, with 2 phr (parts per hundred of resin) content of ZHS. Andre et al. [78] evaluated the smoke suppression property of ZHS and ZS in unsaturated polyester resins combined with halogenated compounds (chlorinated paraffin wax and decabromodiphenyl oxide). The results demonstrated that both ZHS and ZS presented good flame retardancy. The LOI value of the resins containing 10% halogen-based flame retardants showed an enhancement of 2–3 units with a 5 phr content of tin.

Jia et al. [75] synthesized the magnesium-aluminum-iron ternary layered double hydroxide (Mg-Al-Fe-LDH) by the red mud (RM) calcification rehydration method. After coating the surface with ZHS, the coated composites were added into vinyl acetate. The peak smoke emission is significantly reduced, especially since the peak smoke production rate (PSPR) of EVA50/LDH45/ZHS5 is 0.02 m^2^/s, and the average SPR is less than 0.01 m^2^/s, and the PHRR is 219.9 kw/m^2^. Zn^2+^ and Sn^4+^ of ZHS are strong Lewis acids, which can promote the intermolecular crosslinking and carbonization reactions. The resulting char acts as a protective barrier, limiting the diffusion of oxygen to the substrate and retarding the volatilization of flammable products. Additionally, with endothermic decomposition, LDH can release water in its layer structure, thereby cooling the flame zone.

Song et al. [74] used ZHS to coat aluminum phosphate (AlP-ZHS) and then added the composite to PVNVDC, resulting in the uniform dispersion. Active groups (-OH) existed on the surface of AlP-ZHS composites, forming a cross-linked network structure composed of flame-retardant particles and polymer chains. The incorporation of ZHS reduced the PHRR from 167 W/g for pure PANVDC to 142 W/g for ZHS-PANVDC The PHRR value of PANVDC/AlP-ZHS (1:1) composite significantly decreased to 96 W/g, which was due to the shielding effect of the AlP-ZHS composite. In addition, the potential barrier effect of the char layer produced by the AlP-ZHS composite also plays a vital role in hindering heat transfer and gas diffusion. Kim et al. [79] added antimony trioxide (ATO), ZHS, and ATO-ZHS to PANVDC and found that PANVDC-ZHS presented the highest LOI value of 33.5%. The char layer of ZHS-containing PANVDC fibers showed a more stable structure against heat and oxidative carbonization than that of PANVDC fibers and ATO-containing fibers. Lee et al. [80] used a thin material vertical burning test to determine the flame resistance of PANVC fibers with ATO and ZHS. With the increase in the composition ratio of ZHS, the LOI value of PANVC increased significantly, and the LOI value of PANVC increased significantly from 27.9% to 33.1% with 15 phr. Moreover, with the increase in ZHS composition ratio, ZHS promoted dehydroacidification with the formation of PANVC carbonaceous residues, and the carbon layer became denser.

Hamzah et al. [81] used ZHS, calcium borate (CBs), and a non-halogenated flame retardant based on phosphorus/nitrogen synergism (NP-100) as flame retardant fillers in polypropylene (PP) and ethylene propylene diene monomer (EPDM) blends, respectively. After testing and comparison, it was found that after adding 15% flame retardant filler, the composites containing ZHS and CBs showed higher thermal stability than unfilled and NP-100PP/EPDM composites. The results also indicated that ZHS decomposed slowly at a lower rate than NP-100. The addition of ZHS to the PP/EPDM system also reduced smoke production during combustion. This was because their chemical structures included crystalline water, and the water molecules were retained in the CB structure by hydrogen bonding. When the temperature increased, these substances dissociated to release borate and water. The composite material reduced its burning rate by forming char during combustion, thereby protecting the polymer from ambient oxygen. The formation of char was accompanied by the release of water, which diluted combustible vapors. In addition, the char formation reaction was sometimes endothermic which can protect the polymer underlying the char layer. Su et al. [82] prepared PP and polyketone composites with ZHS and intumescent flame retardant additive (IFR). With the addition of 1 wt% ZHS and 24 wt% IFR, the PP/IFR composite passed the UL-94 V-0 rating test with a maximum LOI value of 32%. The TGA data showed that the experimental residue was 6.48% higher than the calculated char yield. Chen et al. [83] utilized machine learning to assist the development of new flame-retardant polymers. Twenty composites were used to train a simple equation for the LOI using the method of sure independence screening and sparsifying operator (SISSO). They also prepared nano-graphene oxide (GO) wrapped micro ZHS to enhance the property of PP. The resulted composites presented enhanced LOI value and V-0 rating in UL-94 vertical tests due to the synergistic effect of ZHS@GO and IFR. 

Wang et al. [20] synthesized uniform ZHS microcubes for suppressing toxic gases (CO, NO_x,_ and HCN) generation of thermoplastic polyurethane (TPU). The presence of ZHS resulted in the reduction in PHRR (−88%) and THR (−50%) via the barrier effect and catalytic carbonization. ZHS also showed a good catalytic degradation of the toxic gases, thus obviously reducing the production of total volatized products and toxic gases such as CO, HCN, and NO_x_.

Zhang et al. [84] synthesized ZHS microencapsulated by aluminum phosphate (AlP) for enhancing the flame retardancy of polylactic acid (PLA). The results showed that the LOI of PLA increased to 32.5% with the addition of 15 wt% AlP/ZHS microcapsule and passed the UL-94 V-0 rating. Additionally, The PHRR and THR were significantly reduced during combustion. Meanwhile, the smoke density (SDR) value reached 44%.

From Table 2, it can be seen that the incorporation of ZHS-based flame retardants leads to a higher improvement in the fire retardant and smoke suppression performance of the polymers. The highest LOI value for PVC can reach 35.5%. However, the ZHS reported so far are all cubic structures with the sharp edge may result in the incompatibility with the polymer chains, which probably reduces the mechanical properties of polymers. In addition, the size of the synthesized ZHS is mostly at the nanometer level, which shows higher flame retardant efficiency than that at the micron scale. More importantly, the synergistic flame retardant and smoke suppression effect between ZHS and other additives have been extensively explored. Among them, ZHS combined with 2D nanoparticles, such as GO, LDH, and g-C_3_N_4_, demonstrates more significant fire and smoke inhibition via an excellent barrier and catalytic effect.

## 4. Prospect

Although ZHS currently shows a good effect on the flame retardant and smoke suppression of polymers, there are still some problems. The preparation of nanoscale ZHS materials exhibits the disadvantages such as complicated preparation process, high product cost, and lack of large-scale application. Since the performance of a single ZHS material cannot meet the requirements of an efficient and stable flame retardant, researchers are required to modify the ZHS material and improve the ZHS performance. In addition, a high addition amount of the currently common ZHS is required to contribute to its flame retardant and smoke suppression effect, which tends to agglomerate and has a certain impact on the properties of the polymer. The emergence of 2D materials, including GO, LDH, and g-C_3_N_4_, may potentially address these issues due to their unique layered structure. Among them, MXenes, transition metal carbides, and/or nitrides, have proven to be an outstanding flame retardant nanofiller, which show an excellent inhibitory effect on the PHRR and THR of polymeric materials [85,86,87,88,89]. More importantly, the additional amount of MXenes is extremely low, often less than 2 wt%. Therefore, the combination of ZHS and MXene may effectively reduce the agglomeration caused by the excessive addition of ZHS. The potential synergistic flame retardant effect between ZHS and MXene is also worth studying.

## 5. Conclusions

This paper outlines the latest results on ZHS-based flame retardancy, including common methods of synthesis of ZHS and its use in flame retardancy of PVC, EP, and other polymers. It indicates that ZHS needs to be synergized with other flame retardants to achieve optimal results. In addition, we present the prospect for the future development of ZHS. It is believed that compounding with 2D materials such as MXene will maximize the flame retardant and smoke suppression properties of ZHS. The amount of the additives can be reduced, further declining the economic costs and avoiding the agglomeration of ZHS without sacrificing the mechanical performance of polymers.

## Figures and Tables

**Table 1:** The comparison of several typical smoke suppression materials.

Materials	Examples	Advantages	Disadvantages
Tin-based	ZnSn(OH)_6_	Great smoke suppression effect; non-toxic	Cubic-shaped ZHS affects the mechanical properties of the polymers
Boron-based	ZnB, boron nitride nanosheets (BNNS)	Non-toxic and environmental-friendly	ZnB shows poor thermal stability and moderate flame retardancy with high loading; BNNS is expensive
Molybdenum-based	MoO_3_	Low content	High cost
Iron-based	Ferrocene	Low toxic	Negative influence on crystallization property of polymer

**Table 2:** Comparison of flame retardant and smoke suppression properties of the ZHS-based polymer composites.

Polymer	Material	Size and Shape of ZHS	LOI(%)	Reduction of PHRR	Reduction of THR	Reduction of TSR	Ref.
PVC	15% ZHS coated CaCO_3_	Cubic-shaped ranged from 1 to 10 μm	35.5	−34.0%	-	-	[58]
	5% DOPO-VTS–ZHS	A diameter of 30–40 nm	30.2	−39.0%	−50.0%	−59.0%	[56]
	5% ZHS/GO	Range of 50–60 nm	28.5	−50.0%	−59.7%	−42.3%	[60]
	2.5% ZHS-TNT	Haw-like structure	29.6	−19.6%	−7.8%	−40.0%	[62]
	10% Sn-4Zn-1CS/rGO	ZHS grain size is larger than that of Sn-4Zn-1CS, up to a grain size of 450 nm	29.7	−36.0%	−24.0%	-	[61]
EP	6% ZHS@NCH	ZHS grain size around 50 nm. Hollow nanocages with nanosheet-constituted shells.	27.2	−69.1%	−14.0%	−36.1%	[63]
	2% ZHS@ Mg-Al-LDH	Cubic structure	25.7	−48.2%	−20.8%	−21.6%	[64]
	2% MnO_2_@ZHS	Cubic-shaped		-	−40.0%	-	[71]
	2% GNS-ZHS-M2070	Several ZHS boxes are deposited on GNS	25.2	−21.8%	−13.4%	−34.6%	[67]
	3% g-C_3_N_4_/β-Ni(OH)_2_/ZHS	ZHS nanoparticles (about 50 nm)	26.2	−39.2%	−15.5%	−14.2%	[68]
	2% AHTSS@PEI@ZHS	The 450 nm solid spheres are uniformly covered by tiny particles	-	-	−29.1%	−33.5%	[70]
	10% CEPPA-ZHS	The diameters of pristine ZHS are 40 nm	24.5	−45.0%	−20.4%	−28.4%	[72]
	3% SBA-15-RGO-ZHS		29.4	−55.0%	−27.0%	-	[66]
	3% ZHS/RGO	Cubic-shaped with an average edge length of around 100 nm	-	−50.3%	−39.0%	−31.0%	[69]
Others	EVA50%/LDH45%/ZHS5%	Cubic-shaped	-	−81.5%	−27.1%	-	[75]
	PANVDC/15%ALP-ZHS (1:1)	a particle diameter of 150 nm to 160 nm	33.2	−42.5%	−7.7%	-	[74]
	PP75%/IFR24%/ZHS1%	50 nm to 200 nm	32.0	−65.0%	26.5%	-	[82]
	TPU85%/APP14%/ZHS1%	The average edge length is approximately 1.5 μm	28.5	−88.0%	−50.0%	-	[20]
	PLA90%/AlP-ZHS10%	AlP/ZHS microcapsule the average diameter was about 700–800 nm	29.5	−61.3%	-	-	[84]

TSR: total smoke release; DOPO: 9,10-dihydro-9-oxa-10-phosphaphenanthrene-10-oxide; VTS: vinyltrimethoxysilane; TNT: Titanium nanotubes; CS: Chitosan; NCH: layered bimetallic (Ni–Co) hydroxides; LDH: layered double hydroxide; GNS: graphene nanosheets; M2070: polyetheramine; PEI: polyethylenimine; AHTSS: amorphous hydrous TiO_2_ solid spheres; CEPPA: 2-carboxyethyl(phenyl) phosphinic acid; SBA-15: mesoporous silica; EVA: Ethylene vinyl acetate; AlP: aluminum phosphate; IFR: intumescent flame retardant; APP: ammonium polyphosphate.

## Data Availability

Not applicable.
